# Inhibiting Invasion into Human Bladder Carcinoma 5637 Cells with Diallyl Trisulfide by Inhibiting Matrix Metalloproteinase Activities and Tightening Tight Junctions

**DOI:** 10.3390/ijms141019911

**Published:** 2013-10-01

**Authors:** Dong Yeok Shin, Hee-Jae Cha, Gi-Young Kim, Wun-Jae Kim, Yung Hyun Choi

**Affiliations:** 1 Dongnam Institute of Radiological & Medicine Sciences, Busan 619-953, Korea; E-Mail: bboglyang@hanmail.net; 2 Departments of Parasitology and Genetics, College of Medicine, Kosin University, Busan 602-702, Korea; E-Mail: hcha@kosin.ac.kr; 3 Laboratory of Immunobiology, Department of Marine Life Sciences, Jeju National University, Jeju 690-756, Korea; E-Mail: immunkim@cheju.ac.kr; 4 Department of Urology, College of Medicine, Chungbuk National University, Cheongju 361-763, Korea; E-Mail: wjkim@chungbuk.ac.kr; 5 Anti-Aging Research Center & Blue-Bio Industry RIC, Dongeui University, Busan 614-714, Korea; 6 Department of Biochemistry, College of Oriental Medicine, Dongeui University, Busan 614-052, Korea

**Keywords:** DATS, invasion, MMP, tight junction

## Abstract

Diallyl trisulfide (DATS), an organosulfur compound in garlic, possesses pronounced anti-cancer potential. However, the anti-invasive mechanism of this compound in human bladder carcinoma is not fully understood. In this study, we evaluated the anti-invasive effects of DATS on a human bladder carcinoma (5637) cell line and investigated the underlying mechanism. The results indicated that DATS suppressed migration and invasion of 5637 cells by reducing the activities and expression of matrix metalloproteinase (MMP)-2 and MMP-9 at both the protein and mRNA levels. DATS treatment up-regulated expression of tissue inhibitor of metalloproteinase (TIMP)-1 and TIMP-2 in 5637 cells. The inhibitory effects of DATS on invasiveness were associated with an increase in transepithelial electrical resistance and repression of the levels of claudin family members. Although further studies are needed, our data demonstrate that DATS exhibits anti-invasive effects in 5637 cells by down-regulating the activity of tight junctions and MMPs. DATS may have future utility in clinical applications for treating bladder cancer.

## 1. Introduction

Bladder cancer is a prevalent malignancy worldwide and the ninth most common type of cancer. It affects three times as many men as women [1,2]. Bladder cancer presents as a non-muscle-invasive, chronically relapsing form, a high-risk non-muscle-invasive carcinoma, or a muscle-invasive form with strong potential to metastasize. High-grade non-muscle-invasive cancers frequently progress, and muscle-invasive cancers are often lethal and have the highest recurrence rate of any malignancy. Although systemic chemotherapy has improved the duration and the quality of life of patients with bladder cancer, long-term survival rates are poor, and the most common cause of mortality is recurrence with metastasis [3–5]. Therefore, development of new agents and more efficient treatment modalities for this disease are needed.

Several epidemiological studies have revealed that dietary intake of *Allium* vegetables is protective against the risk of various types of malignancies including bladder cancer [6,7]. Among *Allium* spp., garlic is a plant commonly used for seasoning food in many different cultures, particularly in Asian countries. As major organosulfur compounds (OSCs) derived from garlic, diallyl sulfide (DAS), diallyl disulfide (DADS), and diallyl trisulfide (DATS) are well-characterized flavor components. These garlic-derived allylsulfides induce cell cycle arrest and apoptosis, as well as inhibit cell invasion by many types of human cancer cell lines. Of them, DATS possesses the most potent cancer activity [8–10]. We have reported recently that DATS-induced apoptosis in human leukemia cells is mediated by the generation of reactive oxygen species (ROS) and subsequent activation of the ROS-dependent caspase pathway [11]. In addition, DATS suppresses cancer cell proliferation and inhibits the growth of transplanted tumor xenografts by inducing apoptosis and/or by blocking abnormal cell cycle progression at the G2/M phase [10,12–16]. However, this effect is selective for cancer cells, as normal cell lines are resistant to cell cycle arrest and apoptosis by DATS [9,12]. Moreover, DATS also inhibits migration and invasion of highly metastatic colon cancer cells via down-regulation of matrix metalloproteinases (MMPs) and inhibition of the nuclear factor-kB signaling pathway [17].

However, no reports address whether DATS affects migration and invasion of human bladder carcinoma cells *in vitro*. Thus, the purpose of this study was to investigate the effects of DATS on cell migration and invasion of the human bladder carcinoma cell line 5637 and to explore the potential mechanisms of the effects. The results indicated that DATS inhibited the migration and invasion of 5637 cells *in vitro* and demonstrated that DATS could serve as a potential anti-metastatic agent.

## 2. Results and Discussion

### 2.1. DATS Inhibits 5637 Cell Migration and Invasion

**Figure 1:**
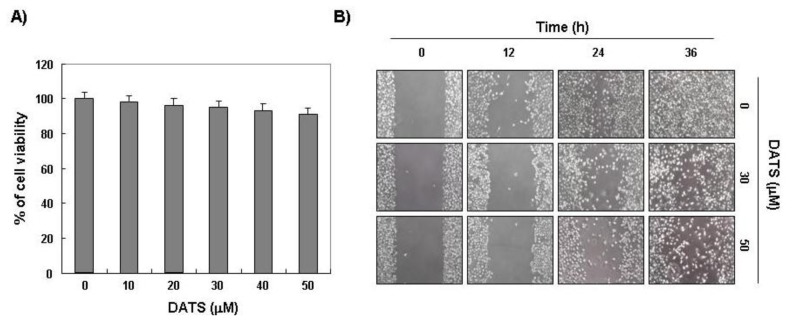
Effects of diallyl trisulfide (DATS) on viability and motility of 5637 cells. (**A**) Cells were seeded at an initial density of 2.5 × 10^5^ cells per 60-mm plate, incubated for 24 h, and treated with the indicated concentrations of DATS for 48 h. Cell viability was measured using an MTT assay. Each point represents the mean ± standard deviation of three independent experiments; (**B**) Cells were grown to confluency on 35-mm cell culture dishes, and a scratch was made through the cell layer using a pipette tip. After washing with PBS, serum-free media (to prevent cell proliferation) containing either vehicle or DATS (30 or 50 μM) was added for the indicated times. Images of the wounded area were taken to evaluate cell movement into the wounded area.

**Figure 2:**
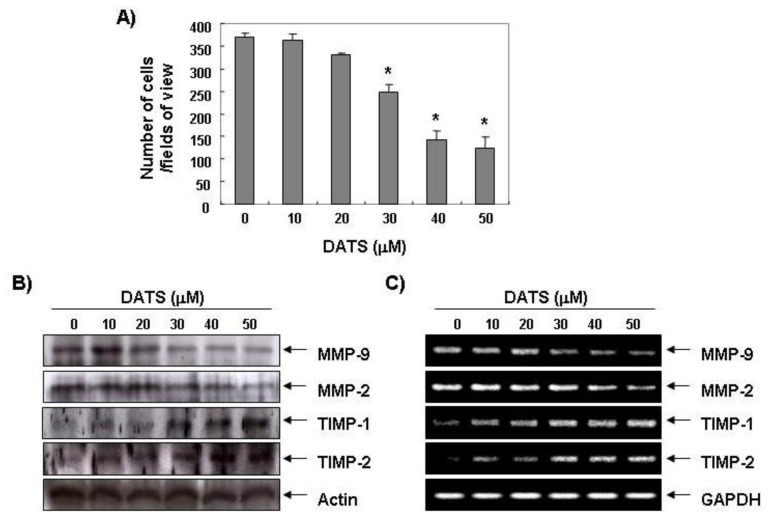
Inhibition of invasion and matrix metalloproteinase (MMP) expression caused by DATS in 5637 cells. (**A**) Cells were plated onto the apical side of Matrigel-coated filters in serum-free medium containing either vehicle or DATS. Medium containing 20% fetal bovine serum was placed in the basolateral chamber to act as a chemoattractant. The invading cells were fixed after 48 h, stained, and then counted. Data are means of triplicate samples and represent invasive cell numbers compared with those of control cells (*p < 0.05 *vs.* untreated control); (**B**) Cells were treated with the indicated concentrations of DATS for 48 h. The cells were sampled, lysed, and 30~50 μg of protein was separated by electrophoresis on sodium dodecyl sulfate (SDS)-polyacrylamide gels. Western blotting was then performed using the indicated antibodies and an enhanced chemiluminescent detection system. Actin was used as the internal control; (**C**) Total RNA was isolated from cells grown under the same conditions as (**B**) and reverse-transcribed. Resulting cDNAs were then subjected to the polymerase chain reaction. The reaction products were run on 1% agarose gel electrophoresis and visualized by ethidium bromide staining. GAPDH was used as the internal control.

Because cell migration is essential for invasion and metastasis of cancer cells, we first examined the effect of DATS on migration and invasion of 5637 cells. A scratch wound healing assay was used for the migration assay. The results showed that DATS significantly inhibited migration of 5637 cells compared to that of the control group in a time- and concentration-dependent manner (Figure 1B). DATS also inhibited cell invasion in a concentration-dependent manner (Figure 2A), as measured by a Matrigel invasion assay. The viability of cells grown under the same conditions was measured by the MTT assay to exclude the cytotoxic effects of DATS on 5637 cell growth. As indicated in Figure 1A, the DATS concentrations used to inhibit cell migration and invasion did not affect cell viability. These results clearly indicate that the inhibition of cell migration and invasion was not due to a cytotoxic action of DATS, and that DATS is an effective anti-migratory and anti-invasive compound in 5637 cells.

### 2.2. DATS Inhibits MMP Expression and Activity

**Figure 3:**
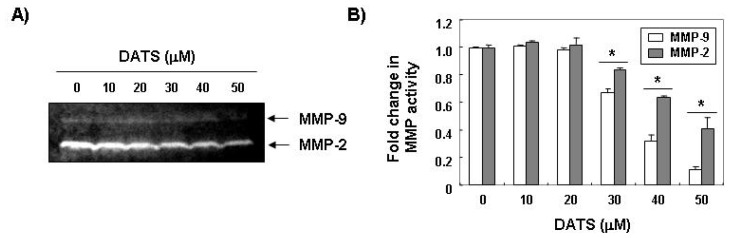
Effects of DATS on MMP activity in 5637 cells. (**A**) The cells were treated with the indicated concentrations of DATS for 48 h. The culture medium was collected and analyzed for gelatinolytic activity by zymography; (**B**) *In vitro* activity of MMP-2 and -9 in cell culture supernatant was measured using a MMP-2 and -9 gelatinase activity assay kit. The biotinylated gelatinase substrates were cleaved by active MMPs in the samples, and the fragments were added to a biotin-binding plate. The digested but unbound fragments were removed by washing. Data are mean ± standard deviation from three independent experiments and are presented as fold change compared with vector control (*p < 0.05 *vs.* untreated control).

One of the most important characteristics of a metastatic cell is the ability to degrade the extracellular matrix (ECM), as this enables the cell to invade through the basement membrane [18,19]. MMPs, a family of zinc-dependent endopeptidases, play a critical role in ECM degradation, which is the starting point of cancer invasion and metastasis. Among MMPs, MMP-2 and MMP-9, which are the gelatinase type of MMPs, promote tumor cell invasion in various cancer cell lines because of their capability to degrade various types of collagens [20,21]. In addition, the activity of MMPs is tightly controlled by transcriptional activation, by a complex proteolytic activation cascade, and by an endogenous system of tissue inhibitors of metalloproteinases (TIMPs). TIMPs inhibit MMPs by forming stoichiometric complexes to regulate matrix turnover [22,23]. Therefore, we determined whether or not the anti-invasive activity of DATS was associated with modulation of TIMP and MMP expression. As shown in Figure 2B,C, DATS markedly inhibited the expression of MMP-2 and MMP-9 mRNA and protein; however, the transcriptional and translational levels of both TIMP-1 and TIMP-2 showed concentration-dependent up-regulation in response to DATS treatment. We next determined whether DATS-induced down-regulation of MMP expression was associated with the inhibition of MMP activities using a zymography analysis and an *in vitro* matrix degradation activity assay. As expected, the gelatinolytic activities of MMP-2 and MMP-9 in 5637 cells decreased significantly following DATS treatment (Figure 3A). Additionally, similar inhibition was observed in the MMP-2 matrix degradation activity assay as well as that for MMP-9 (Figure 3B). Therefore, our results suggest that DATS induced an increase in the TIMP/MMP ratio as a key factor in the regulation of the anti-metastatic process, which subsequently blocked degradation of the ECM and lead to inhibited cell invasion.

### 2.3. DATS Enhances the Transepithelial Electrical Resistance (TER)

**Figure 4:**
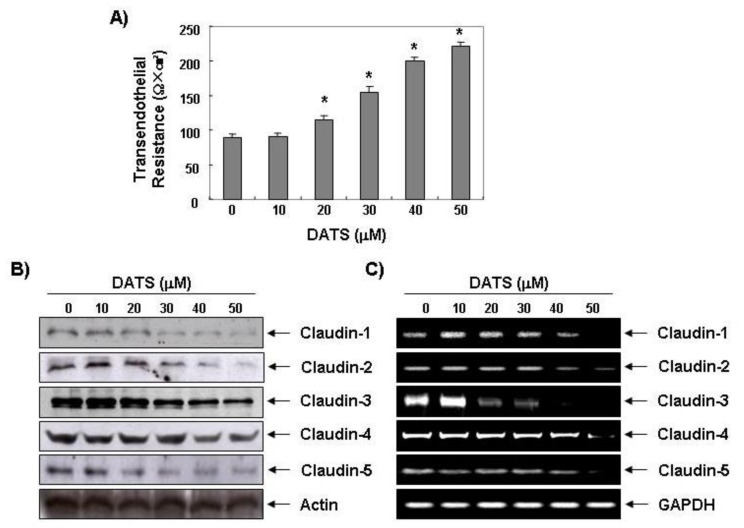
Effects of DATS on Transepithelial Electrical Resistance (TER) values and expression of MMP and tissue inhibitors of metalloproteinases (TIMPs) in 5637 cells. (**A**) Cells were treated with various concentrations of DATS for 48 h, and TER values were measured using an EVOM Epithelial Tissue Voltohmmeter. Results are mean ± standard deviation of three independent experiments. Student’s *t*-test was used to determine significant differences (*p < 0.05 *vs.* untreated control); (**B**) After an incubation with DATS under the same conditions as those of (**A**), the cells were sampled, lysed, and 30–50 μg protein was separated by electrophoresis on SDS-polyacrylamide gels. Western blotting was performed using the indicated antibodies and an enhanced chemiluminescent detection system. Actin was used as the internal control; (**C**) Total RNA was isolated and reverse-transcribed. Resulting cDNAs were then subjected to the polymerase chain reaction, and the reaction products were subjected to 1% agarose gel electrophoresis and visualized by ethidium bromide staining. GAPDH was used as the internal control.

Several specialized and distinct intercellular structures in epithelial cells, including gap junctions, tight junctions (TJ), adherens junctions, and desmosomes are responsible for establishing contact between neighboring cells. These junctions mechanically link normal epithelial cells, and they generate signals that are sent to the interior of the cell to report on the extent of contact with neighbors and the ECM. Another hallmark of malignant transformation of epithelia is that these connections, particularly TJs, are lost [24,25]. In precancerous lesions of the epithelia and cancerous epithelia, TJ strands become disassembled and disorganized or lost altogether, and the TJs cause loss of cell polarity and an increase in motility and invasiveness. Thus, there is an association between the loss of cell-cell adhesion structures and metastasis in many epithelial cancers, as indicated by decreased resistance to electrical current (measured by TER) and increased markers of paracellular permeability. TER values were measured to further examine the relationship between TJ remodeling and the anti-invasive activity of 5637 cells treated with DATS. As shown in Figure 4, incubating 5637 cells with DATS concentration-dependently increased TER, suggesting that DATS increases tightening of the TJs in 5637 cells.

### 2.4. DATS Inhibits Claudin Family Member Expression

Many TJ proteins (claudins, occludins, and junctional adhesion molecules) are linked to the actin cytoskeleton through cytoplasmic accessory proteins to tighten the cell structure and maintain a barrier. Among them, claudin family proteins comprise a multi-gene family of 27 species and are major integral membrane proteins forming the backbone of TJs that are directly implicated in the barrier and adhesive functions of endothelial and epithelial cells [26]. A growing body of evidence reveals the highly contextual up-regulation of claudin expression in certain cancer types, and, moreover, the relevance of claudins in promoting cancer cell invasion, and metastatic progression [27–29]. For example, overexpression of claudin-3 and -4 has been demonstrated in several tumors, including breast and ovarian cancers [30,31]; however, knockdown of these two claudins inhibits cancer cell invasiveness [32]. Yoon *et al.* [33] showed that claudin-1 plays a causal role in acquisition of invasive capacity in human liver cells, which is associated with increased expression of MMP-2. In the present study, we found that DATS concentration-dependently inhibited expression of claudins-1–5 at the transcriptional and translational levels in 5637 cells (Figure 4B,C). However, unlike the result attained in this research, it has been reported that the increase of expression for some types of claudins can promote the increase and spread of cancer cells. For instance, when the expression of claudin-3 or claudin-4 was suppressed, it not only caused the increase and spread of ovarian cancer, but it facilitated an epithelial-to-mesenchymal transition [31,34]. Additionally, there was no significant change in TER, but there was an increased activation in β-catenin signaling or PI3K/Akt signaling. When differentiated embryo-chondrocyte expressed gene 1 (the basic helix-loop-helix transcriptional regulator in breast cancer cell) was knocked down, the invasive capacity increased. It was reported that this is associated with the increase of transcription activation in claudin-1 [35]. Although future studies will need to clarify the precise mechanism and signal transduction pathway for the relationship between the increased TER values and claudin down-regulation, these results demonstrate that the loss of claudins contributes to TJ tightening. Furthermore, the laboratory recently utilized AGS human gastric carcinoma cells and researched the anti-invasive activity of DADS. The OSCs contained two sulfurs [36]. According to precedent research, the suppression of invasion due to the DADS is associated with the increase of tightening in TJs, but the invasion-suppressed effects of cancer cells resulting from DATS was much more effective in the low concentration treatment group compared to DADS. Even though the types of cancer cells used in the two experiments were different, DATS had a more outstanding antioxidant, anti-inflammatory, and cancer cell extinction effect of the three kinds of OSCs. DAS presented as the lowest [9,37,38]. These results signify how the number of sulfur atoms is an important factor that decides the biological activation of OSCs.

In summary, we demonstrated the anti-invasive effects of DATS on 5637 human bladder carcinoma for the first time: (1) DATS inhibited cell migration and invasion; (2) DATS inhibited MMP-2 and MMP-9 activity and expression by up-regulating TIMP-1 and TIMP-2 expression; and (3) DATS increased TJ tightening associated with down-regulating claudin expression. Overall, our data provide a new experimental basis for the clinical application of DATS in cancer treatment.

## 3. Experimental Section

### 3.1. Cell Culture and Growth Inhibition Assay

Human bladder carcinoma 5637 cells were purchased from the American Type Culture Collection (Rockville, MD, USA) and cultured in RPMI 1640 medium supplemented with 10% (*v*/*v*) fetal bovine serum (FBS), 1 mM glutamine, 100 U/mL penicillin, and 100 μg/mL streptomycin (Gibco BRL, Grand Island, NY, USA) at 37 °C in a humidified atmosphere of 95% air and 5% CO_2_. The colorimetric 3-(4,5-dimethylthiazol-2-yl)-2,5-diphenyltetrazolium bromide (MTT) viability assay (Sigma-Aldrich Chemical Co., St Louis, MO, USA) was used to assess growth according the manufacturer’s instructions.

### 3.2. Wound Healing Assay

The 5637 cells were grown to confluence on 35-mm cell culture dishes coated with 20 μg/mL rat tail collagen (BD Biosciences, Bedford, MA, USA) and wounded by scraping the cells with a pipette tip. The cells were then cultured in the presence or absence of DATS in serum-free media for the indicated times. Wound closure of cells was observed and photographed under a microscope at ×40 magnification. Experiments were repeated three times.

### 3.3. Cell Invasion Assay

Matrigel invasion assays were used to assess the ability of 5637 cells to penetrate the ECM in the presence or absence of DATS. Briefly, 5637 cells were cultured in serum-free media overnight. The cells (5 × 10^4^ cells) were loaded on pre-coated Matrigel 24-well invasion chambers (BD Biosciences) in the presence or absence of DATS. Then 20% FBS medium was added to the wells of the plate to serve as a chemoattractant for the cells. The cells in the Matrigel invasion chambers were incubated for 48 h. The invading cells were fixed with 10% formalin, stained with Harris modified hematoxylin and eosin Y (Sigma-Aldrich, St. Louis, MO, USA) and counted (three fields of each triplicate filter) using an inverted microscope [39].

### 3.4. Western Blot Analysis

The cells were harvested, lysed, and protein concentrations were quantified using a Bio-Rad protein assay (Bio-Rad Lab., Hercules, CA, USA), according to the manufacturer’s procedure. Antibodies against MMPs and TIMPs were purchased from Santa Cruz Biotechnology Inc. (Santa Cruz, CA, USA), and those for the claudins were obtained from Invitrogen Corp. (Carlsbad, CA, USA). An antibody against actin was purchased from Sigma-Aldrich. Peroxidase-labeled donkey antirabbit immunoglobulin and peroxidase-labeled sheep antimouse immunoglobulin were purchased from Amersham Corp. (Arlington Heights, IL, USA). An equal amount of protein was separated by 8%–10% sodium dodecyl sulfate-polyacrylamide gel electrophoresis (SDS-PAGE) and then electrotransferred to a nitrocellulose membrane (Schleicher & Schuell, Keene, NH, USA) for Western blot analysis. The blots were probed with the desired antibodies for 1 h, incubated with diluted enzyme-linked secondary antibodies, and visualized by enhanced chemiluminescence according to the manufacturer’s procedure (Amersham, Arlington Heights, IL, USA).

### 3.5. RNA Extraction and Reverse Transcription-Polymerase Chain Reaction (PCR)

Total RNA was isolated from cells using an RNeasy kit (Qiagen, La Jolla, CA, USA) and primed with random hexamers to synthesize complementary DNA using AMV Reverse Transcriptase (Amersham, Arlington Heights, IL, USA). The PCR was carried out in a Mastercycler (Eppendorf, Hamburg, Germany) using primers. Conditions for the PCR reactions were 1× (94 °C for 3 min), 35× (94 °C for 45 s; 58 °C for 45 s; and 72 °C for 1 min), and 1× (72 °C for 10 min). Amplification products obtained by PCR were electrophoretically separated on a 1% agarose gel and visualized by ethidium bromide (Sigma-Aldrich, St. Louis, MO, USA) staining [40].

### 3.6. Gelatin Zymography

The gelatinolytic activities of MMP-2 and MMP-9 in the conditioning culture medium were assayed by electrophoresis on 10% polyacrylamide gels containing 1 mg/mL gelatin at 4 °C. After electrophoresis, the gels were washed in 2.5% Triton X-100 for 1 h and incubated at 37 °C for 24 h in activation buffer (50 mM Tris-HCl, pH 7.5, 150 mM NaCl, 10 mM CaCl_2_, and 0.02% NaN_3_). After staining with Coomassie Blue R-250 (10% glacial acetic acid, 30% methanol, and 1.5% Coomassie Brilliant Blue) for 2 h, the gels were destained with a solution of 10% glacial acetic acid and 30% methanol without Coomassie Blue for 1 h. White lysis zones, indicating gelatin degradation, were revealed by staining with Coomassie Brilliant Blue R-250.

### 3.7. *In Vitro* MMP Activity Assay

MMP activity in the supernatant was measured using the MMP Gelatinase Activity Assay Kit (Chemicon International Inc., Temecula, CA, USA), according to the manufacturer’s instructions. Briefly, aliquots of culture media were incubated with biotinylated gelatinase substrates provided by the manufacturer to cleave active MMP-2 and MMP-9 in the culture media. The fragments were then added to a biotin-binding 96-well plate and incubated for 30 min at 37 °C to allow the biotin-containing fragments to bind to the plate while digestion continued. The digested but unbound fragments were removed by repeated washing, whereas the undigested biotin-labeled gelatinase that bound to the plate was detected by adding a streptavidin–enzyme complex that resulted in a colored product measured at a wavelength of 540 nm with a microplate reader (Molecular Devices, Palo Alto, CA, USA).

### 3.8. TER Measurements

TER (a measure of TJ formation) was measured with an EVOM Epithelial Tissue Voltohmmeter (World Precision Instruments, New Haven, CT, USA), equipped with a pair of STX-2 chopstick electrodes. Briefly, 1 × 10^5^ cells in 0.5 mL of complete medium were seeded into 12-mm Transwell inserts with a membrane pore size of 0.45 μm (Corning Costar Corp., Corning, NY, USA), placed in 24-well plate containing 0.75 mL of growth medium, and allowed to reach full confluence, after which fresh medium was replaced for further experiments. Inserts without cells, inserts with cells in medium, and inserts with cells with DATS were treated. Readings were taken 2 days after seeding the cells. The TER values were calculated by subtracting the blank values from the sample values and then normalizing those values to the growth area of the monolayer.

### 3.9. Statistics

Each experiment was performed in triplicate. Results are expressed as means ± standard deviations. Significant differences were determined using Student’s *t*-test. A *p <* 0.05 was considered significant.

## 4. Conclusions

In this study, we have suggested the mechanism of DATS-induced inhibition of cell motility and invasiveness in human bladder carcinoma 5637 cells. The results indicated that DATS exposure caused inactivation of MMP-2 and -9 by reducing their expressions at both the protein and mRNA levels, and the up-regulation of TIMP-1 and TIMP-2 expression. The increased TJ tightening by DATS treatment was associated with the marked inhibition of claudins, major components of TJs. Although further controlled trials are warranted, these results provide evidence that DATS inhibits the metastasis of human bladder cancer *in vitro*.
